# Tracking and Profiling Repeated Users Over Time in Text-Based Counseling: Longitudinal Observational Study With Hierarchical Clustering

**DOI:** 10.2196/50976

**Published:** 2024-05-30

**Authors:** Yucan Xu, Christian Shaunlyn Chan, Evangeline Chan, Junyou Chen, Florence Cheung, Zhongzhi Xu, Joyce Liu, Paul Siu Fai Yip

**Affiliations:** 1 School of Public Health Li Ka Shing Faculty of Medicine The University of Hong Kong Hong Kong China (Hong Kong); 2 Department of Psychology The University of Hong Kong Hong Kong China (Hong Kong); 3 Department of Social and Behavioural Sciences City University of Hong Kong Hong Kong China (Hong Kong); 4 The Hong Kong Jockey Club Centre for Suicide Research and Prevention The University of Hong Kong Hong Kong China (Hong Kong); 5 School of Public Health Sun Yat-sen University Guang Zhou China; 6 Department of Social Work and Social Administration The University of Hong Kong Hong Kong China (Hong Kong)

**Keywords:** web-based counseling, text-based counseling, repeated users, frequent users, hierarchical clustering, service effectiveness, risk profiling, psychological profiles, psycholinguistic analysis

## Abstract

**Background:**

Due to their accessibility and anonymity, web-based counseling services are expanding at an unprecedented rate. One of the most prominent challenges such services face is *repeated users*, who represent a small fraction of total users but consume significant resources by continually returning to the system and reiterating the same narrative and issues. A deeper understanding of repeated users and tailoring interventions may help improve service efficiency and effectiveness. Previous studies on repeated users were mainly on telephone counseling, and the classification of repeated users tended to be arbitrary and failed to capture the heterogeneity in this group of users.

**Objective:**

In this study, we aimed to develop a systematic method to profile repeated users and to understand what drives their use of the service. By doing so, we aimed to provide insight and practical implications that can inform the provision of service catering to different types of users and improve service effectiveness.

**Methods:**

We extracted session data from 29,400 users from a free 24/7 web-based counseling service from 2018 to 2021. To systematically investigate the heterogeneity of repeated users, hierarchical clustering was used to classify the users based on 3 indicators of service use behaviors, including the duration of their user journey, use frequency, and intensity. We then compared the psychological profile of the identified subgroups including their suicide risks and primary concerns to gain insights into the factors driving their patterns of service use.

**Results:**

Three clusters of repeated users with clear psychological profiles were detected: episodic, intermittent, and persistent-intensive users. Generally, compared with one-time users, repeated users showed higher suicide risks and more complicated backgrounds, including more severe presenting issues such as suicide or self-harm, bullying, and addictive behaviors. Higher frequency and intensity of service use were also associated with elevated suicide risk levels and a higher proportion of users citing mental disorders as their primary concerns.

**Conclusions:**

This study presents a systematic method of identifying and classifying repeated users in web-based counseling services. The proposed bottom-up clustering method identified 3 subgroups of repeated users with distinct service behaviors and psychological profiles. The findings can facilitate frontline personnel in delivering more efficient interventions and the proposed method can also be meaningful to a wider range of services in improving service provision, resource allocation, and service effectiveness.

## Introduction

### Background

In response to the growing demand for accessible mental health support, the number of text-based counseling services has grown exponentially over the past decade [1-3]. Whereas phone-based counseling or telephone counseling services provide mental health support through phone calls or video chats [4], text-based counseling services do so through SMS text messages or emails [5,6]. Similar to telephone counseling services, many text-based counseling services also follow a model of crisis intervention with the intent to provide one-off interventions [7]. The aim of such services is to provide timely mental health support to a wider population, including those who cannot afford consultation fees or are unwilling to speak over the phone.

Several unique features contribute to the popularity of text-based counseling. First, the anonymous nature of text-based communication can reduce the potential stigma that offline services or phone-based services carry [5]. Second, text-based counseling allows users to contact the service and send messages at any time and from anywhere at their own pace, which further reduces their barriers to seeking help [6]. Third, text-based counseling is appealing to people who prefer written communication or have social anxiety [8]. However, new challenges emerge when these services struggle to meet the diverse needs of different types of users, whose service use may vary greatly. While most users may use the service once or just a few times, some might do so frequently. A systematic review of frequent callers to helplines has addressed the concern of inefficient use of service caused by the disproportionate resources taken up by frequent users [9]. In text-based services, frequent users might hinder the effectiveness of the service platforms, as they could overburden the system and reduce other users’ service accessibility [10]. Individuals who frequently reach out for support without any constraints would occupy many sessions and service hours, which can result in extended waiting times for other help seekers, thereby impeding their access to the service. These users may also develop a reliance on the counselor, creating a challenge for both parties involved [11]. Therefore, it is imperative for service providers to understand the varying levels of needs and dependence of their users.

### Frequent Users

In the studies of hotlines and helplines, *frequent users* is a term used to describe those who make significant volumes of calls within a certain period [9]. The characteristics of this group of users have been extensively studied, especially in Australia and the United States. A few demographic characteristics of identified frequent users seem to be consistent across studies. For example, frequent users are more likely to be unmarried, have significant mental health conditions, and be unemployed or have financial difficulties [12-17]. These patterns suggest that, to some extent, users’ frequent and intensive use of the services is associated with some demographic characteristics; that is, there are systematic individual differences in how likely a person would repeatedly use a service.

In terms of suicide risk, although many studies have shown that frequent users are more likely to present suicide ideation [12,17,18], a systematic review suggested otherwise; that is, there was no difference in the level of suicidality between frequent and nonfrequent users [14]. One possible reason for such contradicting results may be the differences in the nature and features of the services. However, the more apparent and plausible reason is that the categorization of frequent users is more often than not ad hoc, arbitrary, and thus inconsistent across studies. The lack of a clear and consistent definition for frequent users poses both theoretical and practical challenges to those seeking to understand and better serve this subgroup of service users.

### Repeated Users

Another term used in the literature is *repeated users*, which refers to those who seek help from helplines or crisis lines more than once [9,18]. Other earlier studies have adopted more restrictive definitions, including inappropriate use [19] or generating multiple calls [20]. The contact pattern of repeated users could vary greatly. Some might return once while others might return daily or monthly. Similarly, some repeated users might frequently contact a service intermittently over a concentrated period, whereas others might do so consistently across a longer time span. Those who use a service occasionally with genuine needs and distress usually do not pose a significant burden to the service providers. Similarly, users who contact the service repeatedly during a certain period and stop when their problems have been resolved are also likely considered a desired outcome for both users and providers. However, some repeated users may become persistent and intensive users who treat these services as long-term support without recognizing the services’ limits or boundaries [15,18]; this type of users can become a significant challenge to the service provider, as they only represent a small fraction of all users while occupying a disproportionate amount of resources [17] at the expense of other nonfrequent users’ opportunity to access the service [18]. As one of the counseling goals is to promote the autonomy and self-efficacy of clients, frequent, on-demand support might become counterproductive in the long run [21].

Many studies have used the terms *repeated users* and *frequent users* interchangeably, rather than distinguishing them according to their service behaviors, characteristics, and help-seeking needs. A systematic review of frequent or repeated helpline callers found that one-third of the studies provided no information on how these groups were defined [9]. Another one-third of the reviewed studies mixed frequent callers with repeated callers, and the remaining one-third used *greater than 8 calls a month* as their definition of frequent users. However, no clear explanation was given for this 8 times-per-month threshold. The mixing of frequent and repeated users as one homogenous group and the lack of a shared understanding of categorization and definition make it impossible to develop standardized strategies to identify and systematically and constructively assist different types of repeated users. Indeed, Spittal et al [17] point out specifically that the arbitrary threshold for the average number of contacts within a moving window cannot adequately reveal the heterogeneity of frequent and repeated users. More information should be considered, including the service journey from the beginning to termination of use, the duration of each session, and use patterns, to distinguish different frequent user subgroups and identify how services might tailor their strategies to meet different users’ needs based on these patterns.

Taken together, there are several research gaps that necessitate a more nuanced classification and understanding of repeated users of hotline and text-line services: (1) most studies of frequent or repeated users focused on telephone-based services, while the profiling in text-based counseling remains relatively unknown; (2) the definition and operationalization of frequent or repeated users are not well-established, as previous studies tended not to differentiate repeated users from frequent users, and most studies classified frequent users based on an arbitrary number of contacts in a period, which neglects the heterogeneity of frequent users; and (3) other aspects of the profiles of frequent or repeated users, including their contact patterns and downstream service satisfaction, have thus far been largely unexplored. To fill these gaps, we first propose to differentiate repeated users from frequent users. We define *repeated users* as those who make >1 contact with the service. In contrast, the term *frequent users* refers to those with heavy use of or dependent behaviors toward the service. Inherently, *frequent users* is a subcategory of *repeated users*. We adopted the term *repeated users* as the master category to investigate service use because it is more inclusive than *frequent users*. Our objective was to develop a systematic way to identify and classify repeated users of text-based services. This would enable service workers to offer targeted interventions to different types of users and improve the service effectiveness. On the basis of previous literature suggesting that suicidal individuals are more likely to be repeated users [17,18,22,23], we hypothesized that compared with one-time users, repeated users require more contacts and longer session duration because they have higher suicide risk and more complicated presenting problems such as addictive behaviors. In addition, we anticipated that suicide risk and severe presenting issues are associated with a higher frequency and intensity of service use.

## Methods

### Participants and Data Collection

Data for this study were extracted from a free 24/7 text-based web-based counseling service called Open Up, which is a free service established to provide round-the-clock support to Hong Kong youth who are aged between 11 and 35 years and are experiencing psychological distress, common mental disorders, and suicidality [10,24]. The platform is staffed by professional social workers and trained volunteers. On average, registered social workers at Open Up have around 8 years of working experience, including 1 to 2 years in web-based counseling. Most volunteers are students with a background in psychology, counseling, or social work. The training information has been documented elsewhere [25]. Users can contact the platform 24/7 via different channels, including the service’s web portal, WhatsApp, Facebook Messenger, WeChat (WeChat channel was terminated in June 2020 due to security concerns), and SMS text messaging. A user successfully connected to the service is assigned to an available staff. After a brief greeting, the service provider applies different approaches to assist the user, including identifying the main issue of concern, showing empathy and care, providing useful suggestions and helpful resources, and referring to offline services if needed. Toward the end of the session, the service provider may refer the user to a follow-up service (either on the web or offline), if deemed necessary.

### Ethical Considerations

This study was conducted under the Education & Knowledge Division of the Open Up project. This study has involved human participants and was approved by the Human Research Ethics Committee at the University of Hong Kong (EA210185; 12/4/2021). Participants provided informed consent to the use of data for research and evaluation purposes before participating by accepting the Terms of Service. Except for the service provided and referrals, participants did not receive any financial incentives. We extracted session text and metadata based on the unique identifier of each user (ie, IP address or social media ID). The unique identifier was first transformed into an 8-digit pseudo-ID before being analyzed. Other private information disclosed during the session was masked to the research team. Finally, this study was conducted with health care professionals of 5 local nongovernmental organizations (The Boys’ and Girls’ Clubs Association of Hong Kong, Caritas Hong Kong, Hong Kong Children and Youth Services, The Hong Kong Federation of Youth Groups, and St. James’ Settlement).

### Procedure

In this study, we included session transcripts and the metadata from those whose first contact was between October 2018 and July 2021 (n=29,400). Users’ first incoming sessions and sessions during the first year (ie, 365 days) were extracted to investigate their contact patterns. We chose to use a 1-year window for several reasons. First, it allowed us to conduct a standardized comparison between users who contacted the service in different periods. Second, the service that we evaluated began in October 2018, and we conducted this study in August 2022, resulting in a data set spanning around 3 years. Using an inclusion cutoff longer than 1 year would exclude more recent data after 2021, as these users would not have qualified to be enrolled. Conversely, using a shorter period may have resulted in insufficient information and use patterns to be investigated. Third, using a 1-year window allowed us to include a larger number of participants, accounting for approximately 92% of the total users of the service. The session metadata, session transcripts, and postsession survey results of the users were extracted for analyses.

**Figure 1:**
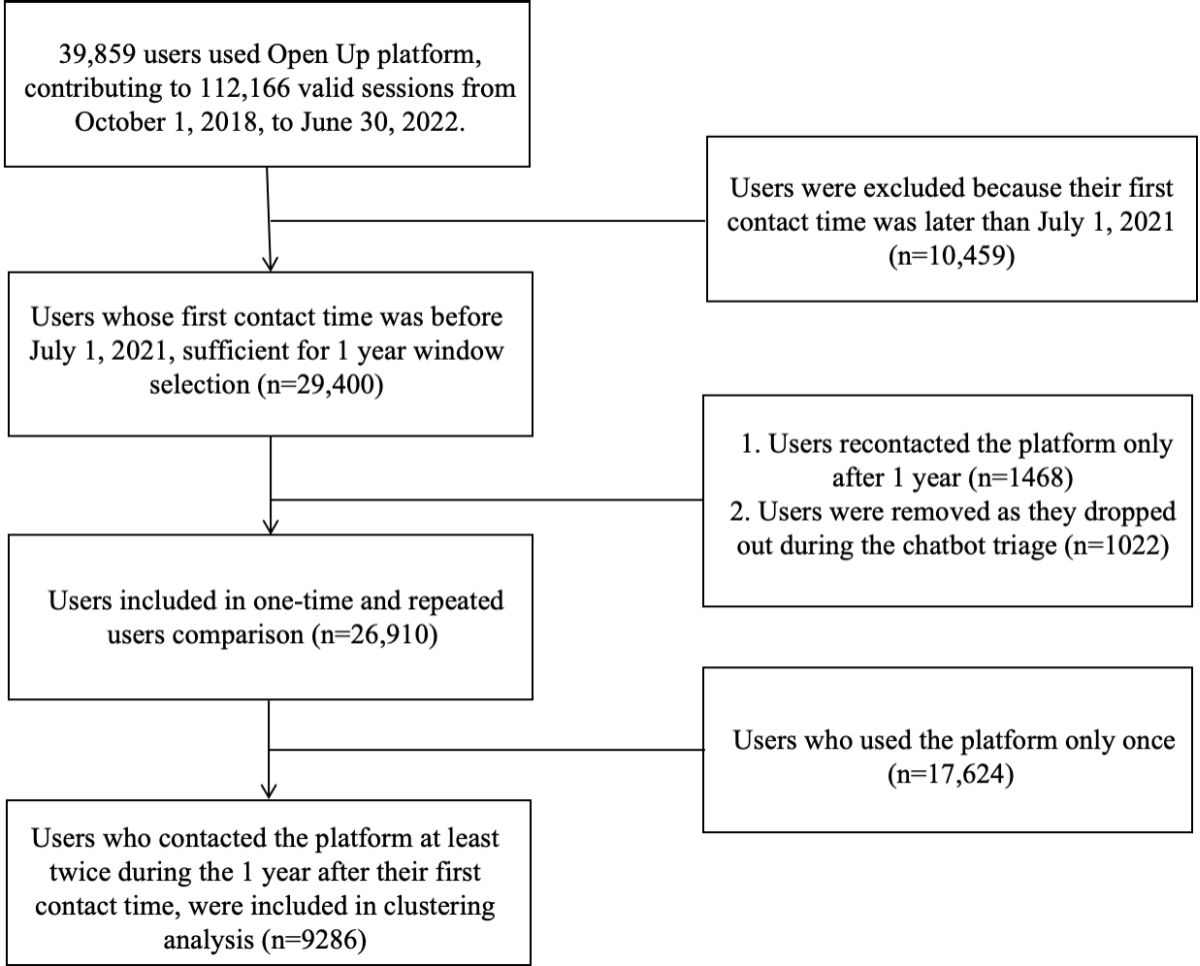
Data inclusion flow.

Figure 1 reports the data included for analysis. A total of 39,859 users used Open Up from October 2018 to June 2022, contributing a total of 112,166 valid sessions (defined by Open Up as those with at least 4 message exchanges between counselors and help seekers). The threshold of 4 message exchanges is based on a previous study that has shown that sessions with <4 message exchanges typically indicate that clients are logging onto the platform without any intention of using the service [25]. Among a portion of the users (n=29,400) whose first contact was before July 2021, which was the cutoff point for the inclusion window at the time of the data analysis, most users (n=26,910, 91.5%) met the inclusion criteria. Those users did not reconnect with the platform only after 1 year or drop out of the service during the chatbot triage process. Consequently, this yielded a sample of one-time (n=17,624, 65.5%) and repeated users (n=9286, 34.5%) over a 1-year period for comparison. The later 9286 repeated users were also included in the clustering analysis.

### User Identification

Open Up is an anonymous and multichannel supported platform. Therefore, the identification of unique users was based on unique identifiers from their incoming platforms, including the IP address for web portal, phone number for SMS text messaging and WhatsApp, and social media account for Facebook and WeChat. No personal identifying information was extracted.

### Operationalization of Repeated Users and One-Time Users

By our proposed definition, users who contacted the platform more than once within 365 days were classified as repeated users, whereas users who contacted Open Up once and did not return within the subsequent 365 days were defined as one-time users (17,624/26910, 65.5%).

### Measures

We evaluated users’ service use behaviors, user profile, and self-reported satisfaction from the postsession survey.

#### Service Use Behaviors

Session metadata collected automatically by the system were used to evaluate users’ service use behaviors. Session metadata includes the date of their contacts, duration of the session, number of message exchanges between counselors and users, and the platforms used (eg, web portal).

#### User Profile

User profile includes demographic information, suicide risk, and presenting issues. Users’ demographic information including age group and gender were extracted from the counselors’ notes if the user disclosed them during the session. Users’ suicide risk levels were assessed by using a 7×5 suicide risk assessment scale derived from previous studies [26,27]; to fit the local context, the scale was modified by 2 counseling experts, a professor of social work and a counseling psychologist, and endorsed by the frontline counselors of the service (Multimedia Appendix 1). The suicide risk assessment scale comprises 7 factors, including 5 risk factors and 2 protective factors. The 5 risk factors include suicide ideation, suicide plan, access to suicide means, suicide action or past attempts, and functioning (ie, physical and psychological functioning such as sleep disturbance and diet problems). The 2 protective factors include the level of perceived social support and use of health or psychosocial support services. Risk factors were assessed using a 5-point Likert scale with scores ranging from 0=no risk to 4=severe risk. Protective factors were rated on a similar but reversed scale. An automated 2-layer process, which was implemented to scan through the session content in real time, first picked up keywords and phrases that belonged to any of the 7 factors, then identified their score based on the keywords list of that factor, and concurrently summed the scores from the 7 factors to provide a total score. The accumulative risk score was then transformed to a risk level based on the rubric, including no risk, low risk, medium risk, high risk, and crisis. *No risk* indicates that the texter does not have any suicidal thoughts. *Low risk* suggests that the texter might occasionally think about suicide but does not have any specific plans. *Medium risk* indicates that the texter might have a suicide plan. *High risk* indicates that the texter not only has a suicide plan but also has access to the means to carry it out. *Crisis risk* signifies that the texter might engage in self-injurious and suicidal behaviors. Counselors were also tasked to check and, if needed, manually modify the risk level based on their clinical judgment. Users’ presenting issues were detected by the keywords and phrases related to different topics. Open Up has 18 predefined topics (based on the early stage of human exploration and annotation of the data), including mental distress, family issues, and intimate relationships (Multimedia Appendix 2).

#### User Satisfaction

User’s postsession satisfaction was assessed to evaluate the service effectiveness, including three questions from the user postsession survey: (1) “How do you feel after the session compared to before?” (2) “Do you find this service helpful?” (3) “Would you recommend our service to your friend?” For the first 2 questions, a 5-point Likert scale was used to specify the level of user’s agreement on the statement, with scores ranging from 1=not helpful at all or much worse to 5=very helpful or much better. For the third question, users responded either “Yes” or “No.” Similar questions were used to evaluate the service effectiveness in comparable services, such as Crisis Text Line [28] and National Suicide Prevention Lifeline [29].

Furthermore, we also included another indicator, premature departure, which refers to dropping out from a session without an agreed closure or an indication of the intention to leave. Premature departure was found to be associated with user dissatisfaction [22,30,31]. Premature departure was detected using the method developed by Xu et al [31].

### Strategies to Classify Repeated Users

**Figure 2:**
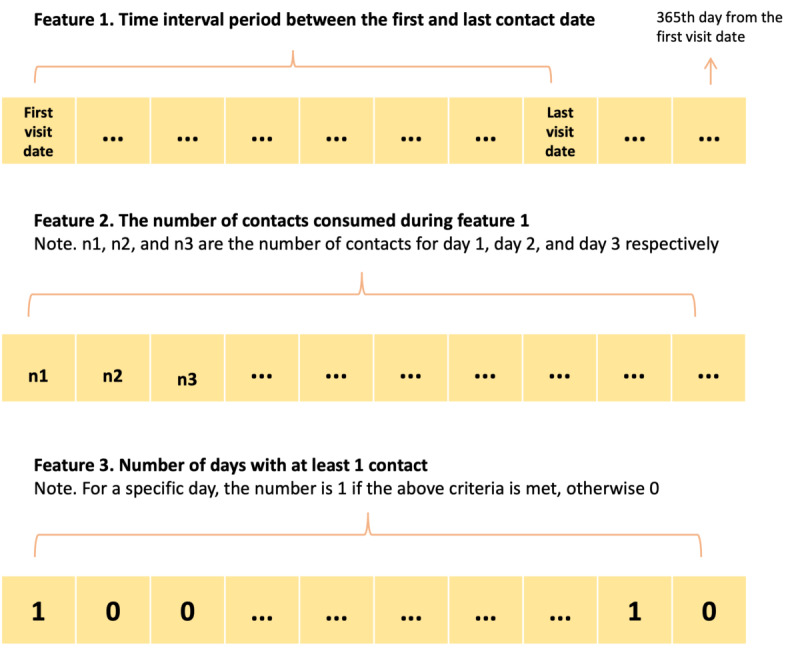
Illustration of the 3 indicators of users’ contacting behaviors.

Instead of using an arbitrary number to define frequent users adopted by previous studies, which inevitably suffers from the loss of interpretability across different frequency levels, we aimed to bridge the research gap by incorporating more features that are indicative of a user’s contact behaviors. The classification of repeated users was based on each user’s 1-year use pattern since their first contact. The clustering analysis used three indicators of each user’s service use (Figure 2): (1) the interval period between the first and last contact date, (2) the number of valid contacts that occurred during the first 365 days, and (3) the number of days with at least 1 contact. We investigated users’ service use based on these 3 indicators because they not only reflect the overall use of the service but also consider the intensity. By considering the user journey in the platform (feature 1), intensity (feature 2), and frequency (feature 3), we can render a more comprehensive picture of users’ help-seeking behavior and classify service users into meaningful groups with distinct profiles and needs.

### Statistical Analysis

As a starting point, descriptive statistical analyses were performed on one-time and repeated users’ first sessions to investigate the reasons why repeated users return and one-time users do not. We decided to use features from their first session for 2 main reasons. First, according to the reviewed studies, users’ contacting behaviors and service use patterns are at least partially associated with their background characteristics. Second, including subsequent sessions from repeated users may introduce confounding factors, such as newly emerged presenting issues, which could potentially influence their characteristics. Hence, focusing on the first session helps to eliminate these confounders and ensures a more rigorous analysis. Subgroup analyses were conducted using chi-square tests to identify the difference regarding user profiles and user satisfaction. Mann-Whitney-*U* tests were used to compare user contact patterns, including session duration, number of message exchanges between counselors and users, and users’ satisfaction after the session. Cramér V (φ_c_) was used to represent the effect size of the chi-square test, while common language effect size was applied to describe the effect size of the Mann-Whitney *U* tests.

Then, we used a bottom-up hierarchical clustering method to systematically classify repeated users into different groups and further investigated the differences between clusters of repeated users. Hierarchical clustering is a clustering algorithm that seeks to build nested clusters by merging or splitting them distinctly in a hierarchical fashion. The root of the structure represents the one inclusive cluster for all samples, and every sample of data in the structure is a cluster itself. The subclusters are merged together at intermediate levels to the root node by their linkage metrics [32]. Hierarchical clustering is widely used for classifying documents in a variety of contexts including language models on unstructured text data [33] and structured electronic health records [34,35], with its advantage of producing meaningful hierarchies and interactive visualizations. In this study, we used hierarchical agglomerative clustering, where each data point starts in its own cluster, and pairs of clusters are merged hierarchically based on the linkage between them [36]. Several linkage algorithms can be used in hierarchical cluster analysis to determine how the clusters are merged. In this study, we used the Ward linkage method, which minimizes the sum of squared differences within all groups. Python package sklearn (Python Software Foundation) [37] was used to perform the hierarchical clustering. A dendrogram was used to visualize and decide the optimal number of clusters, which was decided by the largest vertical gap without any horizontal line passing through the area [38]. The clusters determined by this line are more distinct and separate, as there is more separation space between the clusters. The length of the vertical line represents the distance to be bridged to form the new cluster. Weekly heat maps were used to visualize the use patterns of users from different clusters.

Statistical analyses were performed between different clusters of repeated users regarding user profiles, contact patterns, and user satisfaction. Chi-square test was used to compare the user profiles and satisfaction between clusters, and the Kruskal-Wallis *H* test was used to compare the service use behavior and satisfaction between clusters. Post hoc pairwise comparisons were performed with false discovery rate correction for the chi-square test [39] and with Dunn test for Kruskal-Wallis *H* test [40]. Cramér V (φ_c_) and epsilon-squared (ε^2^) were computed to calculate the effect sizes [41].

## Results

### Hierarchical Clustering

**Figure 3:**
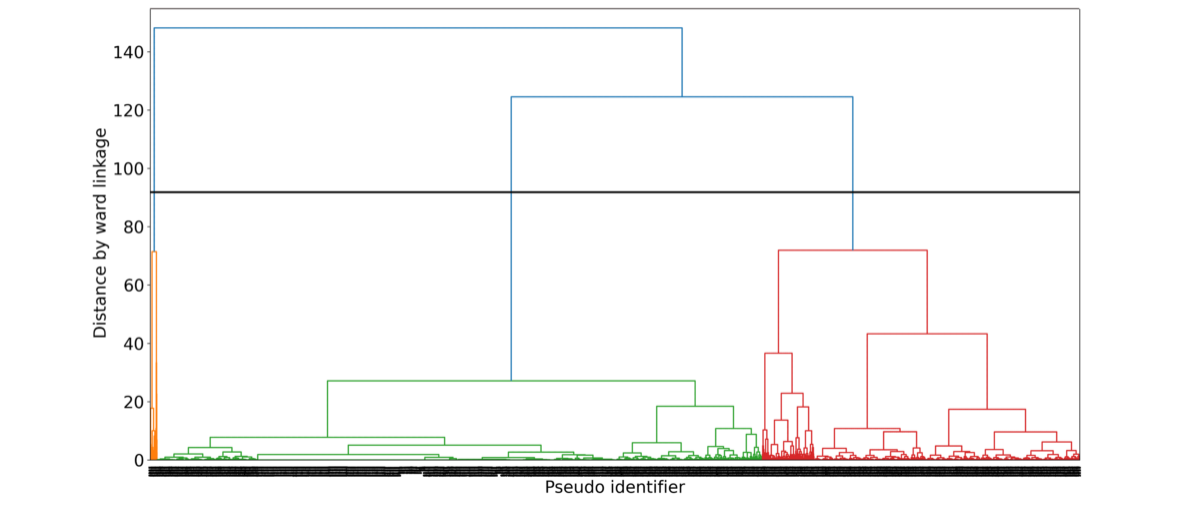
Dendrograms of repeated users with hierarchical clustering. The x-axis represents pseudoidentifiers of users. The y-axis represents the distance by Ward linkage between clusters. Each point (ie, pseudoidentifier) starts with its own cluster, and each point joins other points into a new cluster hierarchically based on the Ward linkage distance until forming the ultimate 1 cluster. Each vertical line represents 1 formed cluster. The length of the vertical line represents the distance to be bridged to form the new cluster. The black solid line represents the cutoff of choosing the optimal number of clusters. The orange, green, and red colors represent different clusters in the final results.

Figure 3 visualizes the results of hierarchical clustering. The algorithm starts by considering each point as a separate cluster and joining points to clusters hierarchically. Each vertical line represents 1 cluster. The cutoff for selecting the optimal number of clusters was visualized as the horizontal line (in black) in Figure 3, which gives us 3 clusters (blue vertical lines).

**Figure 4:**
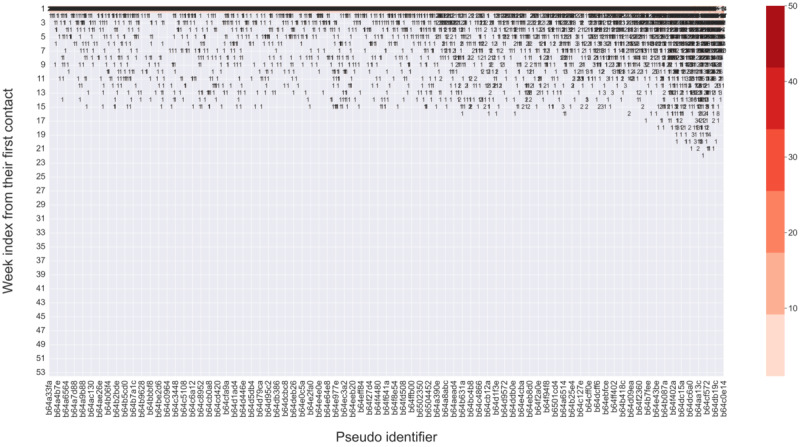
Intensity and contact patterns of episodic users during the 1 year after their first contact (n=6002). The x-axis represents the unique identifier of each user, and the y-axis represents the week index from one’s first contact. The number represents the number of contacts in a particular week, and the shades of color represent the intensity of service use. The figure was sorted in ascending order based on the total number of contacts of each user, from left to right. Consequently, the user identifier on the x-axis indicates a progression in service use from left to right. Only the sampled user identifiers are displayed on the x-axis due to the limitation of the figure size. The dense concentration of numbers during the initial week is attributable to the first-week use of all users in this cluster. As time passed, individual variations started to emerge, and some users dropped out, resulting in a decrease in intensity in the following weeks.

**Figure 5:**
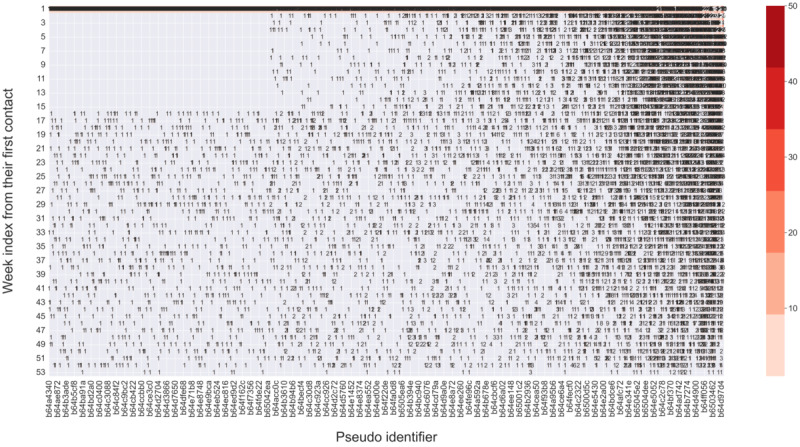
Intensity and contact patterns of intermittent users during the 1 year after their first contact (n=3214). The x-axis represents the unique identifier of each user, and the y-axis represents the week index from one’s first contact. The number represents the number of contacts in a particular week, and the shades of color represent the intensity of service use. The figure was sorted in ascending order based on the total number of contacts of each user, from left to right. Consequently, the user identifier on the x-axis indicates a progression in service use from left to right. Only the sampled user identifiers are displayed on the x-axis due to the limitation of the figure size. The dense concentration of numbers during the initial week is attributable to the first-week use of all users in this cluster. As time passed, individual variations started to emerge, and some users dropped out, resulting in a decrease in intensity in the following weeks.

**Figure 6:**
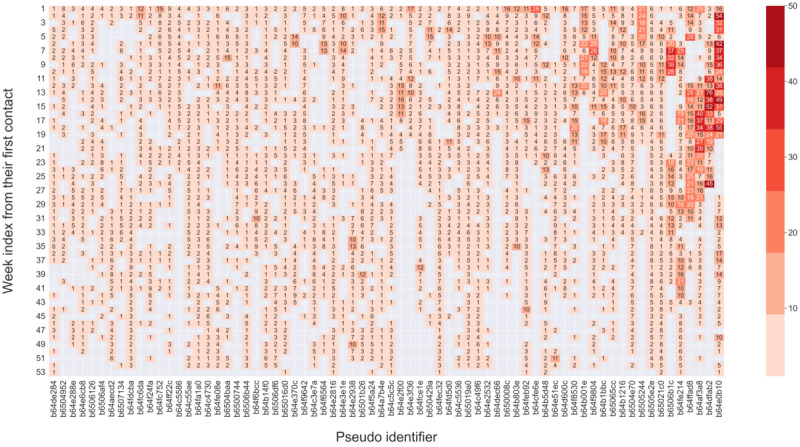
Intensity and contact patterns of persistent-intensive users during the 1 year after their first contact (n=70). The x-axis represents the unique identifier of each user, and the y-axis represents the week index from one’s first contact. The number represents the number of contacts in a particular week, and the shades of color represent the intensity of service use. The figure was sorted in ascending order based on the total number of contacts of each user, from left to right. Consequently, the user identifier on the x-axis indicates a progression in service use from left to right. The dense concentration of numbers during the initial week is attributable to the first-week use of all users in this cluster. As time passed, individual variations started to emerge, and some users dropped out, resulting in a decrease in intensity in the following weeks.

In total, 3 clusters of repeated users were detected. Table 1 reports the basic information about them. They were classified as follows: (1) *episodic users*, accounting for 64.63% (6002/9286) of total repeated users and 36.27% (20,549/56,660) of total sessions among repeated users, only sporadically contacted the platform for the first 2 weeks (median 2 [IQR 1.0-3.0] days of contact); (2) *intermittent users*, accounting for 34.61% (3214/9286) of total repeated users and 44.75% (25,356/56,660) of total sessions, had a long interval of service contact (contact interval, mean 233.6, SD 83.6 days); however, they did not contact the platform too many times, and the number of days with contact was low (median 3 [IQR 2.0-5.0] days); and (3) *persistent-intensive users*, comprising 0.75% (70/9286) of total repeated users and 18.99% (10,758/56,660) of the total sessions, came to the platform nearly every week for the whole year after their first contact, and their median days with contacts were 83 (IQR 71.0-103.5). The intensity and frequency of contact behaviors were different between the 3 groups of users. Figures 4-6 visualize the contact pattern of 3 clusters.

**Table 1:** Contact patterns of the 3 clusters of repeated users.

Cluster category	Total number of contacts, mean (SD)	Interval (days), mean (SD)	Days with contacts, mean (SD)	Total number of contacts, median (IQR)	Interval (days), median (IQR)	Days with contacts, median (IQR)	Users (n=9286), n (%)	Sessions (n=56,660), n (%)
Episodic users	3.4 (3.1)	18.5 (28.5)	2.4 (2.0)	2.0 (2.0-3.0)	3.0 (0-27.0)	2.0 (1.0-3.0)	6002 (64.63)	20,546 (36.27)
Intermittent users	7.9 (11.9)	233.6 (83.6)	6.2 (8.5)	3.0 (2.0-7.0)	234.0 (164.0-309.0)	3.0 (2.0-5.0)	3214 (34.61)	25,356 (44.75)
Persistent-intensive users	153.7 (114.8)	317.8 (69.1)	94.4 (35.5)	115.0 (97.3-146.5)	354.0 (301.3-362.0)	83.0 (71.0-103.5)	70 (0.75)	10,758 (18.99)

### First Session Comparison of One-Time and Repeated Users

Detailed characteristics of users regarding their contact behaviors, profiles, and satisfactions are reported in Table 2. Among the 26,910 users who contacted the platform during the study period, 65.49% (n=17,624) were one-time users, and 34.51% (n=9286) were repeated users. The session use results revealed that the average session duration of the first session was longer among repeated users than among one-time users (cl=0.509; *P*=.02). The number of messages exchanged with counselors was also higher among repeated users than one-time users (cl=0.516; *P*<.001). 64.01% (17,224/26,910) of users revealed their age and gender. There were more female than male users in general and in both the one-time and repeated user groups. Nonstudent youth and middle school students (usually aged 12 to 18 years) and below were more represented than other categories in both one-time and repeated user groups. The web portal was the dominant channel for both one-time and repeated users. However, repeated users used more social media channels than one-time users (φc=0.046; *P*<.001). The risk profile comparison indicated that repeated users showed a higher percentage of high suicide risk and crisis suicide risk sessions than one-time users (φc=0.040; *P*<.001). The results regarding presenting issues revealed that repeated users had more suicide- or self-harm–related concerns, as well as those concerning interpersonal relationships, medical issues, bullying, and addictive behaviors (eg, substance abuse and gambling), than one-time users (φc=0.029, φc=0.023, φc=0.027, φc=0.029, and φc=0.031, respectively; all *P*<.001). By contrast, one-time users had more concerns related to family issues (φc=0.026; *P*<.001), intimate relationships (φc=0.017; *P*=.006), and trending topics (eg, social unrest and COVID-19; φc=0.018; *P*=.004).

As a comparison of service satisfaction, repeated users had a higher rate of premature departure than one-time users (φc=0.065; *P*<.001). The completion rate of the postsession survey was similar across the 2 groups (2965/17,624, 16.82%, among one-time users vs 1960/9286, 14.65%, among repeated users). Among those who filled out the survey, repeated users gave more positive feedback compared with one-time users in all 3 satisfaction questions, including feeling better after the session (cl=0.532; *P*<.001), finding the service more helpful (cl=0.540; *P*<.001), and more likely to recommend the service to others (φc=0.068; *P*<.001).

**Table 2:** Comparing the contact behaviors, profiles, and satisfaction between one-time and repeated users on their first contact (n=26,910).

Variables		One-time users (n=17,624, 65.49%)	Repeated users (n=9286, 34.51%)	Chi-square (*df*)/*U*	*P* value		φc/cl	
**Session duration (minutes)^a^**				83,222,903		.02		0.509
	Values, mean (SD)	55 (38)	57 (43)					
	Values, median (IQR)	47 (27-74)	49 (26-78)					
**Message exchanges**				84,442,795		<.001		0.516
	Values, mean (SD)	38 (29)	42 (34)					
	Median (IQR)	32 (15-53)	33 (15-57)					
**Sex^b^, n (%)**				98.675 (2)		<.001		0.061
	Male	3543 (20.10)	1615 (17.39)					
	Female	8102 (45.97)	3964 (42.69)					
	Unknown	5979 (33.93)	3707 (39.92)					
**Age group^b^, n (%)**				97.174 (4)		<.001		0.060
	Middle school students and below	2618 (14.85)	1405 (15.13)					
	University students	2099 (11.91)	914 (9.84)					
	Nonstudent youth	4766 (27.04)	2266 (24.40)					
	Middle aged	1001 (5.68)	426 (4.59)					
	Unknown	7140 (40.51)	4275 (46.04)					
**Platform, n (%)**				56.762 (4)		<.001		0.046
	Web portal	15166 (86.05)	7691 (82.82)					
	WhatsApp	1314 (7.46)	886 (9.54)					
	Facebook	431 (2.45)	295 (3.18)					
	WeChat^c^	554 (3.14)	305 (3.28)					
	Mobile phone	159 (0.90)	109 (1.17)					
Premature departure, n (%)		7656 (43.44)	4790 (51.58)	161.861 (1)	<.001		0.078	
**Risk level^d^, n (%)**				43.646 (4)		<.001		0.040
	Crisis risk	351 (1.99)	275 (2.96)					
	High risk	731 (4.15)	471 (5.07)					
	Medium risk	6518 (36.98)	3354 (36.12)					
	Low risk	5992 (34.00)	3002 (32.33)					
	No risk	4032 (22.88)	2184 (23.52)					
**Presenting issues^e^, n (%)**								
	Mental disorders or emotional problems	9129 (51.80)	4708 (50.70)	2.911 (1)	.09		0.010	
	Family issue	5660 (32.12)	2744 (29.56)	18.543 (1)	<.001		0.026	
	Intimate relationships	5173 (29.35)	2577 (27.75)	7.536 (1)	.006		0.017	
	Suicide or self-harm	3972 (22.54)	2332 (25.11)	22.324 (1)	<.001		0.029	
	Interpersonal relationships	3789 (21.50)	2185 (23.53)	14.389 (1)	<.001		0.023	
	Study	3601 (20.43)	1926 (20.74)	0.333 (1)	.56		0.004	
	Career prospects or unemployment	2582 (14.65)	1358 (14.62)	0.002 (1)	.97		0.000	
	Medical issue	2235 (12.68)	1355 (14.59)	19.016 (1)	<.001		0.027	
	Work pressure	2234 (12.68)	1124 (12.10)	1.772 (1)	.18		0.008	
	Economic issue	1905 (10.81)	1022 (11.01)	0.221 (1)	.64		0.003	
	Trending topics (eg, social unrest and COVID-19)	1525 (8.65)	708 (7.62)	8.329 (1)	.004		0.018	
	Abuse or sexual assault	1175 (6.67)	676 (7.28)	3.465 (1)	.06		0.011	
	Workplace relationships	1221 (6.93)	654 (7.04)	0.106 (1)	.75		0.002	
	Public examinations	917 (5.20)	558 (6.01)	7.464 (1)	.006		0.017	
	Bullying	780 (4.43)	534 (5.75)	22.688 (1)	<.001		0.029	
	Addictive behaviors (eg, substance abuse and gambling)	610 (3.46)	438 (4.72)	25.273 (1)	<.001		0.031	
	Debt	397 (2.25)	199 (2.14)	0.289 (1)	.59		0.003	
	Sexual orientation or gender distress	364 (2.07)	172 (1.85)	1.309 (1)	.25		0.007	
**Postsession outcomes^f^**								
	Filling rate, n (%)	2965 (16.82)	1360 (14.65)	1.067 (1)	.30		0.006	
	How do you feel after the session vs before?^g^ mean (SD)	3.91 (0.86)	4.01 (0.81)	2,106,417	<.001		0.532	
	Do you find this service helpful?^h^ mean (SD)	3.44 (1.08)	3.60 (1.03)	2,081,828	<.001		0.540	
	Would you recommend our service to a friend in need?^i^ n (%)	1798 (62.98)	880 (68.16)	19.715 (2)	<.001		0.068	

^a^Counselor attend time to last message time.

^b^Extracted from counselor postsession survey.

^c^The use of WeChat was terminated on June 20, 2020.

^d^Suicide risks were scanned by the risk assessment scale.

^e^Presenting issues were detected by keywords and phrases.

^f^n=4325; 2965 versus 1360 for one-time users and repeated users, respectively.

^g^n=4286; 2939 versus 1347 for one-time users and repeated users, respectively (the score ranges from 1 to 5).

^h^n=4235; 2909 versus 1326 for one-time users and repeated users, respectively (the score ranges from 1 to 5).

^i^n=4146; 2855 versus 1291 for one-time users and repeated users, respectively (binary choice).

### First Session Comparison Among 3 Clusters of Repeated Users

A further comparison between clusters of repeated users is tabulated in Table 3. As users’ contact frequency and intensity increased, the average session duration (ε^2^=0.008; *P*<.001) and messages exchanged also increased (ε^2^=0.005; *P*<.001). The percentage of females in intermittent users was significantly higher than in the episodic users (φc=0.039; *P*<.001). Intermittent users also consisted of a different distribution of age groups compared with episodic users, where intermittent users had a higher percentage of middle school students and below, university students, and nonstudent youth (φc=0.038; *P*<.001). As for platform preference, users with more frequent and intensive contacts tended to choose social media platforms rather than the web portal (φc=0.099; *P*<.001). Intermittent users show a lower percentage of premature departure than episodic users (φc=0.073; *P*<.001). The profile comparisons show that users with more frequent and intensive contact behaviors have a higher percentage of high suicide risk and crisis suicide risk sessions than episodic users and intermittent users (φc=0.036; *P*<.001). Contact frequency and intensity were also found to be positively associated with the percentage of users mentioning mental disorders or emotional problems (φc=0.044; *P*<.001). Intermittent users also had a higher percentage of economic issues (φc=0.026; *P*=.048) and study issues (φc=0.028; *P*=.03) and a lower percentage of intimate relationships than episodic users (φc=0.036; *P*=.002). Comparisons in postsession survey questions show no statistical difference between groups of repeated users, potentially owing to the low response rate of the postsession survey.

**Table 3:** Contact behaviors, profiles, and satisfaction between clusters of repeated users^a^ (n=9286).

Variables		Episodic users (n=6002, 64.63%)	Intermittent users (n=3214, 34.61%)	Persistent-intensive users (n=70, 0.75%)	Chi-square (*df*)/Kruskal-Wallis *H* statistics		*P* value		Post hoc or Dunn test		φc/ε^2^	
**Session duration (minutes)^b^**						71.220 (2)		<.001		1<2<3		0.008
	Values, mean (SD)	55 (43)	60 (42)	81 (49)								
	Values, median (IQR)	46 (24-76)	53 (30-81)	73 (48-111)								
**Message exchanges**						47.210 (2)		<.001		1<2<3		0.005
	Values, mean (SD)	40 (34)	43 (34)	58 (43)								
	Values, median (IQR)	31 (15-56)	37 (17-59)	48 (32-77)								
**Sex^c^, n (%)**						28.228 (4)		<.001		1≠2		0.039
	Male	1040 (17.33)	564 (17.55)	11 (16)								
	Female	2459 (40.97)	1466 (45.61)	39 (56)								
	Unknown	2503 (41.70)	1184 (36.84)	20 (29)								
**Age^c^, n (%)**						27.199 (8)		<.001		1≠2		0.038
	Middle school students and below	887 (14.61)	519 (16.15)	9 (13)								
	University students	559 (9.31)	348 (10.83)	7 (10)								
	Nonstudent youth	1414 (23.56)	834 (25.95)	18 (26)								
	Middle aged	274 (4.57)	149 (4.64)	3 (4)								
	Unknown	2878 (47.95)	1364 (42.44)	33 (47)								
**Platforms, n (%)**						181.530 (8)		<.001		1≠2≠3		0.099
	Web portal	5127 (85.42)	2537 (78.94)	27 (39)								
	WhatsApp	491 (8.18)	378 (11.76)	17 (24)								
	Facebook	161 (2.68)	125 (3.89)	9 (13)								
	WeChat^d^	156 (2.60)	136 (4.23)	13 (19)								
	Mobile phone	67 (1.12)	38 (1.18)	4 (6)								
Premature departure, n (%)		3257 (54.27)	1504 (46.80)	29 (41)	49.678 (2)		<.001		1≠2		0.073	
**Risk level^e^, n (%)**						23.483 (8)		.003		1≠2≠3		0.036
	Crisis risk	166 (2.77)	106 (3.30)	3 (4)								
	High risk	291 (4.85)	171 (5.32)	9 (13)								
	Medium risk	2115 (35.24)	1210 (37.65)	29 (41)								
	Low risk	1975 (32.91)	1008 (31.36)	19 (27)								
	No risk	1455 (24.24)	719 (22.37)	10 (14)								
**Presenting issues^f^, n (%)**												
	Mental disorders or emotional problems	2972 (49.52)	1687 (52.49)	49 (70)	17.909 (2)		<.001		1≠2≠3		0.044	
	Family issue	1775 (29.57)	946 (29.43)	23 (33)	0.390 (2)		.82		—^g^		0.006	
	Intimate relationships (lovers)	1737 (28.94)	824 (25.64)	16 (23)	12.229 (2)		.002		1≠2		0.036	
	Suicide or self-harm	1472 (24.53)	837 (26.04)	23 (33)	4.811 (2)		.09		—		0.023	
	Interpersonal relationships	1393 (23.21)	765 (23.80)	27 (39)	9.278 (2)		.01		1=2≠3		0.032	
	Study	1195 (19.91)	714 (22.22)	17 (24)	7.306 (2)		.03		1≠2		0.028	
	Career prospects or unemployment	861 (14.35)	485 (15.09)	12 (17)	1.289 (2)		.53		—		0.012	
	Medical issue	850 (14.16)	487 (15.15)	18 (26)	8.649 (2)		.01		1=2≠3		0.031	
	Work pressure	694 (11.56)	422 (13.13)	8 (11)	4.863 (2)		.09		—		0.023	
	Economic issue	626 (10.43)	389 (12.10)	7 (10)	6.057 (2)		.048		1≠2		0.026	
	Trending topics (eg, social unrest and COVID-19)	445 (7.41)	258 (8.03)	5 (7)	1.141 (2)		.57		—		0.011	
	Abuse or sexual assault	432 (7.20)	238 (7.41)	6 (9)	0.308 (2)		.86		—		0.006	
	Workplace relationships	417 (6.95)	233 (7.25)	4 (6)	0.481 (2)		.79		—		0.007	
	Public examinations	357 (5.95)	196 (6.10)	5 (7)	0.244 (2)		.89		—		0.005	
	Bullying	328 (5.46)	202 (6.29)	4 (6)	2.598 (2)		.27		—		0.017	
	Addictive behaviors (eg, substance abuse and gambling)	264 (4.40)	167 (5.20)	7 (10)	7.342 (2)		.03		1=2=3		0.028	
	Debt	116 (1.93)	82 (2.55)	1 (1)	3.992 (2)		.14		—		0.021	
	Sexual orientation or gender distress	103 (1.72)	69 (2.15)	0 (0)	3.468 (2)		.18		—		0.019	
**Postsession outcomes^h^**												
	Filling rate, n (%)	13.7	16.5	10	13.701 (2)		.001		1≠2		0.038	
	How do you feel after the session vs before?^i^, mean (SD)	3.98 (0.80)	4.06 (0.83)	4.29 (0.49)	6.322 (2)		.04		1=2=3		0.005	
	Do you find this service helpful?^j^, mean (SD)	3.58 (1.03)	3.63 (1.04)	3.86 (1.22)	1.423 (2)		.49		—		0.001	
	Would you recommend our service to a friend in need?^k^, n (%)	70.9	71.5	71.4	5.443 (2)		.25		—		0.046	

^a^Repeated users were detected by unique identifiers, including IP address for web portal, account number for social media channels, and phone number for SMS text messaging.

^b^Counselor attend time to last message time.

^c^Extracted from counselor postsession survey.

^d^The use of WeChat was terminated on June 20, 2020.

^e^Suicide risks were scanned by the risk assessment scale.

^f^Topics were detected by keywords and phrases.

^g^Not applicable.

^h^n=1360; 824 versus 529 versus 7 for episodic, intermittent, and persistent-intensive users, respectively.

^i^n=1347; 819 versus 521 versus 7 for episodic, intermittent, and persistent-intensive users, respectively (the score ranges from 1 to 5).

^j^n=1326; 803 versus 516 versus 7 for episodic, intermittent, and persistent-intensive users, respectively (the score ranges from 1 to 5).

^k^n=1291; 782 versus 502 versus 7 for episodic, intermittent, and persistent-intensive users, respectively (binary choice).

## Discussion

### Implications for Web-Based Counseling Services

In this study, we analyzed the service use and postsession satisfaction of users of a text-based web-based counseling service to identify the potential characteristics of different clusters of users. By examining 3 key indicators of users’ service use, including the interval between the first and the last contacts, the total number of contacts, and the number of days with at least 1 contact, we identified 3 groups of repeated users: episodic users, intermittent users, and persistent-intensive users. Generally, compared to one-time users, repeated users showed higher suicide risks and more complicated backgrounds, including more severe presenting issues such as suicide or self-harm, bullying, and addictive behaviors. Higher frequency and intensity of service usage were also associated with elevated suicide risk levels and a higher proportion of users citing mental disorders as their primary concerns. The findings are potentially beneficial for developing efficient interventions tailored to the specific needs of different repeated user groups and improving service effectiveness.

Data from Open Up showed that 65.5% (17,624/26,910) of users were one-time users, and 34.5% (9286/26,910) had used the service more than once (ie, repeated users). The ratio is similar to other text-based services such as Crisis Text Line (63.8% and 36.2%, respectively) in the United States [42]. Contrary to telephone helplines, where the percentage of male individuals was higher for repeated users than for one-time users, we did not find a significant gender difference between one-time and repeated users [14,15]. Comparing data from their first sessions provides insights into why repeated users tend to recontact the service. Repeated users displayed higher suicide risk levels and disclosed more topics of concern related to suicide or self-harm, interpersonal relationships, medical issues, bullying, and addictive behaviors than one-time users in their first session, implying that they generally had more complicated needs that may require more attention and support, which likely and understandably drove them to use the service again. In addition, repeated users had a more positive first-session experience than one-time users. The fact that they were more satisfied with the service might also motivate them to return. The satisfaction rate of one-time users was on average lower than that of repeated users; some users of the former group did not find the service useful, and perhaps, as a result, these users did not seek help again from this service. This may imply that there is a degree of heterogeneity within the one-time user group. Future research could explore qualitatively and quantitatively the differences among different concerns, such as how counselors handle different presenting issues, user-counselor dynamics, user satisfaction, and rate of premature departure.

In total, 3 clusters of repeated users with unique characteristics were identified. The episodic users display the least service use. Their sessions were shorter in duration and lower in number of exchanges. Episodic users had the lowest suicide risk among the 3 subgroups of repeated users. Their primary concerns tended to be related to intimate relationships. While it might be the case that these users were able to adequately address or resolve those issues in the few sessions, the postsession results show that their satisfaction with the first contact was lower than the other 2 groups of repeated users, which indicates another possibility that they subsequently did not find the service to be helpful, that the sessions did not help them make progress, or that they did not match their expectations. Intermittent users used the service over long intervals but in relatively low intensity. Their risk level was higher than that of the episodic users but lower than that of the persistent-intensive users. The motivation for their use of Open Up could be periodical stressors, such as study, work, and economic stress; they would use the service as needed. Persistent-intensive users are likely the ones that impose the greatest burden on frontline service providers and the system. This group, while small in size (70/9286, 0.75%), accounted for 18.99% (10,758/56,660) of service capacity among repeated users and 14.48% (10,758/74,284) of the total service capacity. Similar to previous studies on telephone helplines, persistent-intensive users tended to present more mental health concerns [13,17]; higher suicide risk [17]; and, perhaps as a result, evidently had longer session time and more contacts to meet their needs. Persistent-intensive users had more concerns about medical issues and addictive behaviors (eg, substance abuse and gambling), which tend to be more chronic and difficult to address. This might lead them to return to the service frequently and intensively. In addition, persistent-intensive users were more likely to experience interpersonal relationship problems, which is found to be one of the main issues that drive suicide ideation [43]. Their higher suicide risk, complicated presenting issues, and exposure to long-term stressors could jointly drive their high frequency and intensity of service use. Preference for different service platforms also helps explain the service use of persistent-intensive users. They tended not to prefer using the anonymous web portal; rather, they generally accessed the service using social media platforms or SMS text messaging services that revealed their phone numbers. It is possible that they were in greater need and looking for a more long-term counseling service instead of a one-off session, and thus, they did not mind disclosing their social media accounts or phone numbers.

From the user postsession satisfaction surveys, there was an observable trend and pattern in the mean scores across the 3 subgroups of repeated users, which may explain why persistent-intensive users (ie, the most satisfied group) returned most frequently. However, due to the low response rate (ie, around 11%), the results were not statistically different. The demographical differences across the 3 categories suggest that there were individual differences that may help explain users’ use of the service. However, the effect sizes of the characteristics were small, and thus, their implications should be considered with caution. Simultaneously, the lack of strict time limits and the idiosyncratic response patterns between different counselors may have intensified the dependent behavior of some users. Text-based network analysis or in-depth qualitative analysis is needed to examine whether there are structural or systematic differences between the ways in which the subgroups were served. Some standardization of service protocols could be considered to improve service effectiveness.

Service capacity and resource allocation are highly influenced by repeated users. The average session duration for persistent-intensive users was >1 hour (mean 81, SD 49 minutes), which was around 50% longer than that of one-time users (mean 55, SD 38 minutes). The capacity taken up by persistent-intensive users should be considered in the service capacity management plan to strategically improve service efficiency and limit users’ unlimited use. Several suggestions could be considered for persistent-intensive users. Since they prefer social media channels more than the anonymous web portals, service providers could arrange scheduled sessions with the same counselors (eg, when the incoming traffic is less busy), minimize their random access to the system (ie, to change the reinforcement schedule from random to fixed), collectively set specific goals per session, and track progress. This will also reduce the need for users to repeat their presenting issues every time they access the service and thus improve service efficiency. We also found that persistent-intensive users displayed higher risk and experienced more long-term stressors than intermittent and episodic users. Therefore, when serving persistent-intensive users, service providers could consider allocating them to more experienced counselors with skills that can cater to the specific issues of concerns. Regular case studies and workshops should also be conducted to equip service workers and update their knowledge and skills. In addition, it was observed that persistent-intensive users displayed a higher proportion of mental disorders or emotional problems, interpersonal problems, substance abuse, and medical problems. While this study did not identify the comorbidity profile of each individual, that is, whether the same user had experienced mental disorders, interpersonal problems, substance abuse, and medical problems or a different combination of these issues, we speculate that some of them might have some degree of personality dysfunctionality [44]. This suggests that intervention strategies for personality disorders, such as modules in dialectical behavior therapy, schema-focused therapy, or mentalization-based treatment, could potentially be helpful to those clients [45]. However, whether these strategies can be effectively delivered in a web- and text-based context such as the current one warrants further investigation [46]. Alternatively, when persistent-intensive users are willing to identify themselves, counselors can refer them to longer-term counseling services or offline services that might better suit their needs. They can also set more structured and standardized time limits. This could potentially be one of the optimal intervention solutions because by doing so, they could prioritize other users that can benefit more from their low-intensity service, as well as reduce persistent-intensive users’ overdependence on the platform without denying or excluding them from service.

At the time of writing in November 2023, ChatGPT and other natural language processing models have attracted growing interest in the field of health care [47] and mental health [48]. There are at least several possible ways that artificial intelligence (AI) models can enhance the efficacy of service workers and support their service provision to help repeated users. In the presession stage, AI models can help triage users by detecting their mental health states and help-seeking issues based on sentiments and linguistic patterns. By doing so, AI models can help prioritize users and provide appropriate support. During the session, AI models can collect and analyze users’ information in real time, assisting in the discovery of effective treatment and intervention strategies. This real-time analysis can greatly aid service workers in providing timely and personalized care. After the session, AI models can review the complete session history of repeat users, aiding service workers in developing a comprehensive understanding of these users’ needs and progress. However, before applying AI models in the service, potential issues including data privacy and security, ethical consideration, integration, and supervision should be considered.

This study has several methodological contributions adding to the existing evidence. First, we used features including total number of contacts in a year, number of days with contacts, and time length between the first contact and the last contact to differentiate repeated users. We see this as an advancement from the prior practice of using an arbitrary number of contacts over a period to categorize frequent users. This approach also allows each service to define its own threshold based on actual use, as services can differ greatly in terms of their targeted clientele, nature, service protocol, and available resources. Second, previous studies used a top-down definition to identify frequent users, which varied across studies and thus made comparisons across studies difficult. To solve this issue, we proposed a bottom-up, data-driven method to classify users, supplementing the current definitions and providing a more objective perspective on understanding frequent and repeated users. Third, our method of classifying repeated users is transparent and straightforward. This can help improve the reproducibility and generalizability of our results and thus aid its application to other counseling or health services. Most importantly, the proposed method successfully identified 3 clusters with distinct service use patterns. This approach can benefit related services in clustering their service users, providing support for their intervention strategy for different types of users, and optimizing their resource allocation. Further in-depth investigations and interventions on different groups of repeated users are needed to identify the best practice for handling different types of frequent users.

### Limitations

This study has several limitations. First, repeated users were identified based on their unique identifiers, which has limitations. IP addresses are not fixed even for the same location. Furthermore, a user may connect to the service from home, school, or work, resulting in multiple IP addresses for the same user. Moreover, the same IP address can be shared by multiple users, for example, an IP address from a school or a cafe. The same user can also create several social media accounts or have different phone numbers, making it impossible to distinguish the user without analyzing the session content. The current analytical practice respects and maintains the privacy and anonymity of the service users. To reduce the noise of data while maintaining user anonymity, future studies could explore the use of digital footprints to match repeated users’ sessions for a fairer comparison.

Second, we did not investigate the long-term behavioral pathways for repeated users. Instead, we focused our analyses on the traits of their first contact and the factors that could predict their return. Future studies can use users’ longitudinal sessions to investigate the changes in dependent behaviors.

Third, while the proposed methods provide a holistic view of repeated users that can facilitate frontline workers for better service provision, it is important to recognize that people who share similar psychological profiles and use patterns may not experience mental illness in the same way. The proposed method cannot provide a one-size-fits-all solution. Therefore, tailored assessment and treatment plans are necessary on a case-by-case basis. Future studies should not neglect the heterogeneity of users from the same cluster with varying experiences of mental illness.

Fourth, due to the low response rate of the postsession survey (4325/26,910, 16.07%), the analysis of between-group differences in satisfaction might be biased and may limit the generalizability of this study’s findings. Low response rates to postservice surveys are not uncommon. For example, Crisis Text Line reported that only 23% of all text conversations were followed by a completed postconversation survey [42], and 9% was reported in another web chat service developed for youth mental health [49]. Also, studies have found that those who opted to fill out a postservice survey tended to be also more satisfied with the service [50,51]. This adds biases to the results. Future studies can seek to improve the response rate of postsession surveys and, as an alternative approach, apply text mining to identify relevant factors to indicate user satisfaction.

### Conclusions

Adequately serving repeated users is one of the most challenging aspects of web-based counseling and crisis intervention services. In this study, we used a bottom-up method based on features of service use to identify the heterogeneity of repeated users. Three groups of repeated users with distinct patterns of service use and risk profiles were identified. The findings can be meaningful to the frontline services in better allocating resources and building service model to improve efficiency and effectiveness. More in-depth analyses of one-time users and repeated users are needed, especially to develop best practices in handling most persistent-intensive users as well as to understand the different reasons why some users do not return.

## Appendix

Multimedia Appendix 1 Risk assessment scale.

Multimedia Appendix 2 Presenting issues detected in Open Up.

## Abbreviations

AI: artificial intelligence
