# Supplementary material for: Tracking and Profiling Repeated Users Over Time in Text-Based Counseling: Longitudinal Observational Study With Hierarchical Clustering
Source: J Med Internet Res. 2024 May 30;26:e50976. doi: 10.2196/50976 (PMC11176871; doi:10.2196/50976)
Supplement: Multimedia Appendix 1 [file jmir_v26i1e50976_app1.docx]

| **Date: / / 2020** | | **Shift:** | | **Counselor:** | |
| --- | --- | --- | --- | --- | --- |
| **Please rate the following items in a 5-point scale.**   \| **4 ––––– 3 ––––– 2 ––––– 1 ––––– 0** \| \| --- \| \| **Crisis High Medium Low No** \| | | \| ***Scores at Total*** \| ***Recommended action*** \| \| --- \| --- \| \| 21-28 (or score 4 on Item 4 **AND 3 or above on Item 1)** \| Crisis - require immediate and active crisis intervention \| \| 15-20 \| High - require close monitoring and follow-up by Open Up at least in the next 72 hours; other professional follow-up services are also recommended \| \| 6-14 \| Medium - require monitoring and referring to other professional follow-up services \| \| 1-5 \| Low - Require regular monitoring \| \| 0 \| No - Require no monitoring and follow-up \| | |  |  |

**Please circle the box that can best describe the case in the recent months (within the past three months): 請於每一評估項目圈出最能描述最近幾個月（過去三個月內）情況的方框:**

Suicide Risk Assessment for Open Up (OUSRA-7) *(May 13, 2020)*

| **Assessment Items** | **0** | **1** | **2** | **3** | **4** |
| --- | --- | --- | --- | --- | --- |
| 1. **Suicide Ideation** (Intent to die)   **自殺念頭**（自殺的念頭） | No suicide ideation  沒有自殺念頭 | Occasional thoughts of suicide  偶然有自殺念頭 | Repeated thoughts of suicide  反覆有自殺念頭 | Frequent but not intensive thoughts of suicide  經常但不強烈的自殺念頭 | Intensive and enduring thoughts of suicide  強烈和持續的自殺念頭 |
| 1. **Plan of attempt suicide** (A plan may include ideas, specific and logical execution steps, feasibility, timing, and preparation)   **自殺計劃**（計劃可以包括想法、具體及有邏輯的行動步驟、可行性、時間、準備措施等） | No plan  沒有自殺計劃 | Plan without details  初步自殺計劃/沒有細節 | Plan with some details  有自殺計劃/並有一些細節 | Concrete plan with preparatory behaviors  有自殺計劃並有準備措施 | Concrete plan with execution steps  有具體自殺計劃並有明確行動步驟 |
| 1. **Means** (Access to and lethality of the means)   **工具** (獲取/接觸工具的途徑和工具的致命性) | No access to means  沒有接觸到工具 | Limited access to means/means may not be lethal  有可能但未接觸到工具 / 工具未必致命 | Access to means / means may be lethal  能獲取工具 / 工具有可能致命 | Access to lethal means or with knowledge of using the means in a lethal way  能獲取工具和懂得怎樣達到致命的效果 | Immediate access to lethal means which can be used at any time  已獲取並且隨時可以使用具致命性的工具 |
| 1. **Action** (Non-suicidal self-inflicted injury / suicide attempts)   **行動** (曾經或正在非自殺式自我傷害／企圖自殺) | No history  沒有嘗試過/沒有記錄 |  | History of non-suicidal self-injury (NSSI) / previous suicide attempt(s)  曾經有非自殺式自我傷害行為／企圖自殺 | Suicide attempt(s)  in the past 3 months  最近(約三個月) 曾經企圖自殺 | In action of self-injury/ suicide  正在非自殺式自我傷害／企圖自殺 |
| 1. **Functioning** (physical & psychological functioning – e.g. sleep disturbance, diet problems, personal hygiene, physical discomfort, chronic pain/illnesses, emotional distress, depressive mood, cognitive impairment, mental disorders, social isolation and/or failure to keep up with daily routines, such as work, school, household chores, or paying bills etc.)   **功能**（生理和心理功能，例如睡眠困難、飲食問題、個人衛生、生理不適、長期痛徵／慢性疾病、情緒困擾、抑鬱情緒、認知障礙、精神病、社交孤立、不能處理日常事務，例如上班、上學、家務、支付帳單等） | No signs of impairment  沒有障礙 | Occasional signs on physical and/or psychological impairment  偶然出現生理和/或心理障礙 | Repeated signs on physical and/or psychological impairment  反覆出現生理和/或心理障礙 | Frequent signs on physical and/or psychological impairment  經常出現生理和/或心理障礙 | Enduring signs with severe impairment on physical and/or psychological functioning  生理和/或心理障礙持續並且嚴重地影響日常生活 |
| 1. **Social support**   (Perceived level of satisfaction about the size, nature, frequency and duration of social support)  **社交支援**（對於社交支援的大小、性質、頻率和時間的滿意程度） | Satisfied with the social support received so far  對於所獲得的社交支援滿意 | Somewhat dissatisfied with the social support  對於所獲得的社交支援有點不滿意 | Dissatisfied with the social support  對於所獲得的社交支援不滿意 | Very dissatisfied with the social support  對於所獲得的社交支援十分不滿意 | Extremely dissatisfied with the social support  對於所獲得的社交支援極度不滿意 |
| 1. **Use of health/psychosocial support service(s)** (Access to and use of service; helpfulness)   **健康／社交心理支援服務的使用**（對於支援服務的接觸和有助程度） | Access to health/ psychosocial support services when needed. Find the service useful  有接觸受健康／社交心理支援服務及認為服務有幫助 | Occasional difficulty accessing to health / psychological service but find them helpful  接觸健康／社交心理支援服務偶遇困難但認為服務有幫助 | Difficult access to health / psychological services but find them somewhat helpful  接觸健康／社交心理支援服務有困難但認為服務有些幫助 | Difficult access to health / psychological services and find them non-helpful  接觸健康／社交心理支援服務有困難及認為服務沒有幫助 | Resistant to receive any of the suggested health / psychosocial support services  不願接受任何健康／社交心理支援服務 |
| **Total** |  |  |  |  |  |
| **Overall assessment and remarks** |  |  |  |  |  |
