# Supplementary material for: Tracking and Profiling Repeated Users Over Time in Text-Based Counseling: Longitudinal Observational Study With Hierarchical Clustering
Source: J Med Internet Res. 2024 May 30;26:e50976. doi: 10.2196/50976 (PMC11176871; doi:10.2196/50976)
Supplement: Multimedia Appendix 2 [file jmir_v26i1e50976_app2.docx]

Appendix 2. Presenting issues detected in Open Up

| **Presenting issues** |
| --- |
| Mental disorders/Emotional problems |
| Family issue |
| Intimate relationships |
| Suicide/Self-harm |
| Interpersonal relationships |
| Study |
| Career prospects/Unemployment |
| Medical issue |
| Work pressure |
| Economic issue |
| Workplace relationships |
| Trending topics (e.g., social unrest, COVID) |
| Abuse/Sexual assault |
| Public examinations |
| Bullying |
| Addictive behaviors (e.g., substance abuse, gambling) |
| Debt |
| Sexual orientation/Gender distress |
